# At the Edge of Viability: Moral and Ethical Guideline Proposals*

**DOI:** 10.5041/RMMJ.10067

**Published:** 2011-10-31

**Authors:** Asa Kasher

**Affiliations:** Department of Philosophy, Tel Aviv University, Tel Aviv, Israel

**Keywords:** Edge of viability, human dignity, moral and ethical guidelines, negative compelling reasons, positive compelling reasons, principle of moral gradation

## Abstract

The paper proposes moral and ethical guidelines for medical treatment at the edge of viability. The proposed principles are defended on the grounds of a general conceptual framework presented by elucidating the notions of viability, the edge of viability, person, sanctity of human life, dignity, and the slope of dignity protection, as well as the distinction between ethics and morality.

## INTRODUCTION

The purpose of the present paper is to suggest moral and ethical guidelines of treatment at the edge of viability, on the basis of a general conceptual framework for discussion of medical cases that involve babies at the edge of viability. The first part of the paper elucidates some of the major concepts. The second part of it outlines and defends general principles as grounds for making decisions with respect to neonates at the edge of viability.

## CONCEPTUAL FRAMEWORK

Naturally, the first notions that need elucidation are those of *viability* and *the edge of viability*. Later a notion of *sanctity of human life* will be added. We will now discuss each of them in turn and several other ones as well.

### Viability

Literally, the notion of *viability* is related to the major capabilities of existence and development. Hence, when viability is attributed to a state of a fetus, what is literally asserted is that the fetus is capable of living and growing in the normal way.

However, the notion of *viability* when applied to a state of a fetus has gained a different sense. Roughly speaking, it refers to the capability to live outside the mother’s womb. When applied to a state of a neonate, *viability* means, again roughly speaking, the capability to live, with no commitment to the quality of the expected life or development of the neonate.

The notion of *capability to live*, as applied to a fetus or a neonate, has to be understood as having the sense of capability to live, albeit with help, even if it is help crucial for staying alive.

A question arises as to whether *viability* means capability to live for any period of time, or rather capability to live for a long period of time. As the notion of *viability* is usually used with respect to a fetus or a neonate, it seems that capability to live, for a rather long period of time, is meant.

Again, one may ask what exactly is meant by a *long period of time.* It seems advisable to respond by introducing two notions of *viability*: First, there should be a notion of *ordinary viability*, which means capability to live, when there are no grounds for predicting a life expectancy of less than average. Secondly, there should be a parametric notion of *t-viability*, where *t* is a certain period of time (months, years, decades). The *t-viability* notion will be usable when there are grounds for predicting life expectancy in terms of *t*. Accordingly, arguments concerning the fate of a fetus or a neonate that are commonly phrased in terms of *viability* should be rephrased in terms of either *ordinary viability* or *t-viability*. We will use the former notion of *viability* unless usage of the latter is specified.

If we combine the observations that have just been made, then we get the following elucidation of the notion of *viability* that will serve us subsequently. An attribution of *viability* to a fetus or a neonate is an attribution of a present capability to live outside the womb, for a long period of time, albeit with some crucial external help. The length of the time involved varies.

So far, we have considered attributions of *viability* as if it is a matter of certainty: as if there is a “yes” or “no” answer to the question as to whether a certain neonate, under certain conditions, is viable or not. When we have a highly corroborated theory, on grounds of which it is possible to make highly accurate predictions, our answers to such particular viability questions can reach a level of near certainty. In practice, probabilities of viability play a role in decision-making procedures. The common extent of uncertainty does not, however, change the definition or elucidation of *viability.*

### Edge of Viability

Given such notions of *viability*, certain general claims can be made about a neonate. For example, it can be claimed that under present medical conditions, a healthy neonate is ordinarily viable if it is born after 25 weeks’ gestation. General claims of that form can rest on grounds of viability statistics or of embryological theory. Since both data and theory are grounds for different viability claims, the natural question arises as to the minimal gestational age of neonates for which it is claimed that if a healthy neonate is of that age or older, then it is viable. Thus, for a given body of knowledge that includes both viability statistics and corroborated theories, the edge of viability is definable. It is the minimal age for which it can be claimed, on the grounds of that body of knowledge, that if a neonate is of that gestational age or older, then it is viable.

Strictly speaking, what has so far been defined as *the edge of viability* is *the edge of viability, relative to a body of knowledge*. Such a body of knowledge includes not only statistics and theories (“knowledge that”), but also methods of medical treatment intended to save life and enhance viability (“knowledge how”). Evidently, since both our “knowledge that” and our “knowledge how” never cease growing, the related edge of viability keeps moving. Claims that the edge of viability is 22 weeks are either already correct or will sooner or later become correct.

### Viability and Edge of Viability

At this point, we would like to point out the nature of these two notions. Attribution of viability or of being at the edge of viability to a certain neonate is a function of three types of knowledge:

Firstly, particular “knowledge that,” which is a specification of the medical conditions of the neonate under consideration. Secondly, general “knowledge that,” which is statistical knowledge or theoretical knowledge pertaining to neonates under the same or similar conditions. Thirdly, “knowledge how,” which is the medical and technological means available for treating that neonate.

Thus, there is no room for any societal decision as to what viability is or what should be considered as the edge of viability. Clearly, specification of medical conditions, statistical data, and scientific theories is outside the confines of societal deliberations and decision-making procedures. Availability of medical and technological means of treatment might seem different, since societal decisions can be made as to whether some means are to be put at the disposal of medical staff for treatment of neonates at the edge of viability or neonates in general. However, such understanding of availability would be misguided. Attribution of viability or being at the edge of viability depends on the facts with respect to the relevant family of methods of treatment, medications, pieces of equipment, etc., in general, and not to any section of it that has been designated by some societal decision of whatever nature. Problems of expenditure give rise to societal deliberations and decisions, but the sharp distinction between matters of fact and matters of societal undertaking of expenses should not be blurred.

### Person

We move now to a brief elucidation of a notion of *person*, which is going to play a major role in our consideration. We are going to elucidate *a* notion of *person* rather than *the* notion of *person*. As a matter of fact there is a common usage of the term *person* neither in daily parlance nor in philosophical literature.

It has often been claimed that the English term *person* was derived from the Latin term *persona*, which meant a mask as used by an actor in a performance. Hence, to use the term *person* in some context of utterance is to ascribe a certain role within that context. Thus, for example, in a legal context, to describe someone (a man or a woman) or something (a company or a state) as a *(legal) person* is to ascribe the legal role of having a cluster of legal attributes, such as duties and rights.

Since we are presently interested in moral and ethical considerations, we are going to elucidate a notion of *person* that is used to ascribe a moral or an ethical role. The difference between a moral context and an ethical one will be explained below. For the present purpose, suffice it to make three assumptions about these contexts:

Firstly, both of them involve normative considerations, namely ones that involve considerations of what is the case but also and mainly of what ought to be the case. Secondly, both of them involve principles and arrangements that can justify claims as to what ought to be the case, under certain circumstances. Thirdly, in both of them the role played by a *(moral) person* or an *(ethical) person* is that of a focus of rights and duties that rest on certain normative principles and arrangements.

On the background of these assumptions, it makes sense to ask who is a person, or, in other words, what are necessary or sufficient conditions for being a person, in a moral or an ethical context.

Philosophical literature provides us with various answers. Here is one list of personhood elements, where reproduction issues are under consideration. The list included: minimum intelligence, self-awareness, self-control, a sense of time, a sense of futurity, a sense of the past, a capability of relating to others, concern for others, communication, control of existence, curiosity, change and changeability, balance of rationality and feeling, idiosyncrasy, and neocortical functioning.

A list often looks arbitrary. Delineation on grounds of a seemingly arbitrary list seems invalid. Our delineation of persons, in a moral or an ethical context, will, therefore, rest on a method of delineation that can be justified as such.

In answering the question, who is a person, in a moral or an ethical context, we proceed along the following line of argument: Firstly, we specify some ingredients of moral and ethical contexts. Secondly, we point out some necessary or sufficient conditions for being able to play a role in a context that has those ingredients. Thirdly, we draw distinctions between conditions that obtain in a full-fledged manner and those that obtain to a lesser extent. Among the latter we draw a distinction between those that are sufficiently similar to the former ones and those that are not. Fourthly, on the grounds of those distinctions we suggest an approximate answer to the question who is a person, in a moral or an ethical context.

We turn now to a brief presentation of these four steps.

Moral and ethical contexts manifest several distinct features. First of all, they involve values and norms, that is to say, claims or arguments related to what is desirable, from some perspective, or what ought to be the case, from some point of view. Then, moral and ethical contexts involve societal arrangements that embody certain values and norms. Finally, such arrangements determine certain distributions of rights and duties that bind their participants.

In the second step of our method, we look for necessary or sufficient conditions for being a participant in those moral or ethical arrangements, which embody values and norms, distribute rights and duties, and are binding. Presently we will confine ourselves to a brief presentation of a few examples. A necessary condition for being a party to a binding arrangement, such as a contract, is the cognitive condition of having a temporal self-identity. That is to say, one’s view of oneself is that of a temporal being of a nature that enables one to identify one’s present stage with some of one’s future stages and both as stages of the same being. Another necessary condition for being a participant in an arrangement that embodies values is the mental condition of having some relatively fixed desires that regulate one’s behavior to a significant extent. Yet another necessary condition for being a participant in an arrangement that distributes rights that are justifications of claims is the cognitive condition of having the abilities to justify a claim, to identify interests, and to remember states of affairs that give rise to claims and their justifications. Similar examples abound.

Usually, healthy adult human beings seem to have all those necessary abilities. Hence, as a second approximate delineation of persons, in a moral or an ethical context, we have the class of those beings that have all those necessary abilities. This class includes all healthy adult human beings.

When we look at some of these abilities, such as the ability to identify certain families of states or the ability to justify certain statements, it becomes clear that a significant distinction should be drawn between different states of ability. On the one hand, we have ability in its most mature form, while on the other hand we have the same ability at an earlier stage of its development, before it becomes mature. Children are our prime examples of human beings who do not have all those necessary abilities, but nevertheless are considered to be parties to the societal arrangements involved in moral and ethical contexts. They are often parties who are different in their distribution of rights and duties, for example, but parties to those arrangements they seem to be all right. Notice, furthermore, that children as well as adults sometimes lack some ability without thereby being put outside the confines of the class of persons, in moral and ethical contexts.

Accordingly, we have now as the third approximate delineation of persons, in moral and ethical contexts, the class of those beings that have all, or almost all, necessary abilities, whether in a mature form or in a well-developed but not yet mature form. This class includes human adults and children, though not all of them.

Finally, we draw a distinction between two developmental stages of ability. To grasp the leading idea underlying the distinction we are going to introduce, consider some ability that involves both a rich genetic endowment as well as a significant process of maturation. Linguistic ability is, indeed, a prime example. Consider, furthermore, the developmental stage of that ability upon birth. The genetic infrastructure of the ability is well developed, to an extent that enables the neonate to start undergoing a process of growth, development, and maturation. The distinction we now introduce is between a developmental stage in which we have a well-developed infrastructure of some ability and a developmental stage of that ability in which the infrastructure is not yet well developed.

Now, our philosophical claim is that during a developmental stage in which the infrastructure of an ability is well developed, a significant part of that ability is already extant. Put differently, during such a developmental stage the ability is already extant, though in a significantly non-mature form.

Most interesting are the “edge” states, the first ones during which the infrastructure is already well developed. The most conspicuous example of our distinction is seen when those “edge” states are compared to the immediately preceding ones. Our empirical assumptions are two: First, that the infrastructures of the above-mentioned necessary abilities reside in the cortex, and, secondly, that the cortex is well developed from the 30th week of gestation and on. If a safety zone is added for accommodating individual differences, we reach the 26th week of gestation as the lower edge of the developmental stages during which the necessary abilities are presumably already extant.

We have now reached the fourth and presently final approximate delineation of persons, in a moral or an ethical context. From a conceptual point of view, what we have as the class of persons, in those contexts, the class of those beings that have all, or almost all, necessary abilities in one of the three following forms: 1) In a mature form; 2) in a well-developed but not yet mature form; and 3) in the form of a well-developed infrastructure of the ability. From an empirical point of view, this means that presumably every adult, child, baby, neonate, and fetus during the third trimester of gestation are persons, in a moral or an ethical context.

### Sanctity of Human Life

Several conceptions govern prevalent attitudes towards the life of a human being, be it a fetus or an adult, and since they are significantly different from each other, we briefly clarify them.

The most entrenched form of adherence to a conception of the sanctity of life is found in the religious tradition of Jainism, which includes respect to all forms of life and a related strict insistence on non-violence. The sanctity of human life is, then, an interesting consequence of a general principle of sanctity of life.

Another conception of sanctity of life is confined to “higher” forms of life, including human life as a prime example. One feature that “higher” forms of life share with each other is sentience. A conception of the sanctity of “higher” forms of life rests on an ascription of “intrinsic value” to what manifests high intricacy of structure and functioning. Such forms of life are “good” as such. Human life is “good” in the same sense. Moreover, what is intrinsically “good” should be well protected. Such an attitude of sanctity of human life serves as both a manifestation of the view of the intrinsic value of human life as well as an instrument of protection of human life.

A narrower conception of sanctity of “higher” forms of life applies to those that manifest, for example, both sentience and rationality. Although such conceptions are not proposed and defended as conceptions of the sanctity of human life, since their notion of “higher” forms of life applies in our era and environment only to human beings, they serve as “edge” conceptions of the sanctity of human life. All other conception will be, in some sense, narrower than that one.

One such conception is found in the tradition of the Jewish religion. Within such a tradition, any conception of “sanctity” applies in a primary sense only to the deity. Every attribution of “sanctity” to human life is, therefore, derivative or even undesirable. However, on the background of some conception of the relationships between the deity and human beings, human life is extremely precious, on the one hand, but is not absolutely protected, on the other hand. According to an ancient Jewish norm, if a Jew is threatened with death unless he commits idolatry, bloodshed, or adultery, then he should be willing to be killed rather than commit such activities. On any other occasion, protection of human life “overrides everything,” every other norm of religious validity. Strictly speaking, this is a conception of the “preciousness,” rather than “sanctity,” of human life, in a somewhat restricted sense.

Finally, we have a “preciousness” conception of human life that does not rest on any view of the intrinsic value of human life or of a divinely endowed value of human life. This conception rests on the simple observation that being alive is a precondition for being a participant in societal arrangements that embody values and norms and distribute rights and duties. (To be sure, it is not meaningless to speak, for example, about the rights of the deceased, but such figures of speech are not paradigmatic.)

According to this conception, protection of human life is protection of what is a necessary element of any valuable societal arrangement. No wonder that from a democratic point of view, the life of the citizen is of uppermost importance. In a sense, this is a “necessary precondition” conception of human life. In the following, we will argue on the grounds of such a conception, which fits every democratic setting.

### Dignity

Protection of human life is one dimension of protection of human dignity. Whereas we clearly have a working understanding of human life and its protection, it seems to be advisable to elucidate some notion of *human dignity* and its protection.

A conception of *human dignity* involves, first of all, a sharp distinction between human beings and objects. Accordingly, protection of human dignity requires practical adherence to that basic distinction by means of clear and regular manifestations of commitment to practices that do not treat human beings on a par with objects, such as stones, cars, computers, and robots, as well as non-human sentient beings.

Secondly, every conception of human dignity allows drawing distinctions between different human beings and even expressing them in our practices. We all treat our loved ones in ways that are very significantly different from the ways we treat all the rest. Our states treat citizens and non-citizens quite differently. However, a moral conception of human dignity sets a practical threshold of respect to be paid to every human being as such.

Thirdly, a moral conception of human dignity puts respect for human autonomy at the center of respect for human beings. To respect a human being is practically, first and foremost, to enable that human being to create, maintain, and nurture a form of life in which one manifests one’s values and taste, beliefs and desires. No wonder, then, that a democracy, which embodies in its form of regime a moral conception of human dignity, has a constitutional bill of rights that protects the autonomy of every citizen in one’s most fundamental spheres of life.

We have outlined a conception of what has been described as *human dignity and its protection*. Actually this is a conception of the dignity of a person (in a moral or an ethical context) and its protection. The elements that have been mentioned apply not only to an adult human being but also to a fetus during the third trimester of gestation.

### The Slope of Dignity Protection

Protection of an area can be done at its gates and borders, without anything being done outside its confines. However, effective protection involves protection both of the area itself and some of its adjacent areas. Protection of the area itself will be of a different nature from that of an adjacent area. Whereas attempts to eliminate any hostile activity within the area itself is constitutive of the very idea of protection of the area, attempts to eliminate or at least weaken hostilities in adjacent areas is an auxiliary method, meant to face a potential danger before it turns into an actual one.

The nature of essential protection is, then, different from that of auxiliary protection, but both kinds are required in a practical system of effective protection.

A major ingredient of auxiliary protection of the dignity of persons, in a moral or an ethical context, is the conception of the slope of dignity protection. Imagine a slope: At the top end of it we have a person, whose dignity is protected. The dignity and its protection are full-fledged. At the bottom end of the slope we have objects that are not the subject matter of any claim for dignity and its protection. A slope is extended from the bottom end to the top end. The higher up something is on the slope, the more dignity it deserves and the more extensive should be its protection. A fertilized egg is above an unfertilized egg, on the slope, but under a blastocyst. A fetus is above an embryo, on that slope, because it is closer in its structure and functioning to a person than an embryo. Protection of the dignity of a fetus is stronger than that of an embryo, in the sense that stronger arguments are required for justifying, say, an abortion of a fetus than an abortion of an embryo.

We will call our duties to protect the life and dignity of a person *primary* ones and the duties to protect the life or dignity of what is on the slope of dignity but not a person *secondary* ones.

A similar slope of dignity protection is involved in human attitudes towards cadavers, bones, and graves. At the top end of the slope, one step removed from the living person, we have the corpse of a person who has just died. At the bottom end we have, say, a grain of sand on a beach. A grave is above that grain of sand, on the slope. A cadaver is above a grave, on that slope, because it is closer to the person in the causal chain of states after death.

Mastering practices of paying gradual respect along those slopes of dignity protection is, therefore, a precondition of successful maintenance of any effective practice of paying respect to persons, who are at the top ends of those slopes.

### Morality and Ethics

Finally, we have to draw a distinction between *moral* and *ethical* contexts. During the history of philosophy, theology, law, and other adjacent areas, those notions have often been used interchangeably. We would, however, like to draw a distinction between two perspectives, using the terms *moral* and *ethical*.

When we witness an interaction between two human beings, we can depict it in different ways. First of all, it can be depicted as an interaction between two persons. Our descriptions of them will be confined to the fact that each of them is a person. However, under ordinary circumstances, the same interaction is amenable to a variety of richer descriptions, e.g. between a parent and a son, a physician and a patient, a social worker and a patient’s relative, to mention just a few examples. Such a description of the same interaction between two persons brings into the picture roles (parent, son, patient) and professions (physician, social worker), among other positions. To bring into the picture a profession, for example, is to bring into it values and norms that regulate proper activity within that profession. An interaction between a person who is of a certain profession and another person can, then, be analyzed and evaluated from the point of view of those values and norms of the profession. The same holds for other positions.

The distinction one should be aware of is that between such different points of view. On the one hand, we have what we will call the *moral* point of view, which evaluates interactions between persons qua persons. Principles of human dignity protection reflect the moral point of view. On the other hand, we have what we will call an *ethical* point of view, which evaluates interactions between persons of certain roles, such as professions. A conception of parental duties, a Code of Medical Ethics and Rules of Conduct for a Company Director reflect ethical points of view.

## MAJOR MORAL AND ETHICAL PRINCIPLES

On the background of the previous section we move now to a brief proposal and defense of principles for treatment of neonates at the edge of viability. Similar principles hold for fetuses at the edge of viability, but they are beyond the scope of the present paper.

### Principle of Moral Gradation

The moral status of a neonate at the edge of viability depends on whether it is already a person more than it depends on its viability.

This principle rests on several of the above-mentioned observations. The primary moral status is that of a person. The moral status of what is not a person but rather very similar to a person is a secondary one. Therefore, the first step in a moral evaluation of an attitude towards a neonate is answering the question as to whether it is a person or not. Under ordinary circumstances, the answer to this question depends on the gestational age of the neonate, i.e. whether it has reached its 26th week. A fetus or a neonate of that age is presumably a person. That is to say, if we do not have compelling reasons for assuming it does not have a well-developed cortex, we consider it to be a person and have to treat it accordingly.

If under consideration is a neonate at the edge of viability, which presently means younger than 25 weeks, then its moral status is not primary but secondary. As far as the neonate of 20-odd gestational weeks is under consideration, there are no moral rights that are infringed if its life or dignity is not protected, since only persons have moral rights. However, we do have secondary moral duties with respect to such a neonate, since we encounter it at a very high point on the slope of dignity protection.

To be sure, we may also be under a primary moral duty to protect the rights of the mother and the father of such a neonate. We shall see later what are those primary moral duties as well.

### Principle of Presumed Medical Duties

Presumably, a neonate at the edge of viability ought to be medically treated on a par with a neonate who is a person.

This principle too rests on observations made during the previous section. The principle of moral gradation draws a distinction between a neonate who is a person and neonates that are not, whereas the principle of presumed medical treatment puts them on a par with each other; they are compatible with each other.

Under ordinary circumstances, a 22- to 25-week-old fetus or neonate is located very high on the slope of dignity protection. First of all, though in some important respects such a fetus or neonate is different from a 26- to 30-week-old fetus or neonate, they are in many respects quite similar to each other. Moreover, the process of transformation from the earlier stage to the later one is, under ordinary circumstances, natural and relatively short. Hence, the secondary moral duty to respect the dignity of the viable neonate, during the former stage, cannot induce a form of protection that would be very different from the form of protection enjoyed during the latter stage. The difference between them cannot be of taking care, to the best of the medical ability, of the life and health of the neonate after the edge of viability, and not giving any medical attention to the neonate at the edge of viability. The most appropriate form of respect for the dignity of what is very similar to a person would be the form of respect for the dignity of a person. Any other form would involve an unacceptably steep slope.

How can one follow both the principle of presumed medical treatment, which requires respect of a certain form, and the principle of moral gradation, which seems to require the opposite? The answer rests on the first word of the principle of presumed medical treatment, i.e. *presumably*.

A presumption reflects a burden of proof. The familiar presumption of innocence reflects a clear distribution of the burden of proof: The prosecution has to prove that the defendant is not innocent, and if it fails in its attempts to do so the defendant does not have to do anything in order to prove his innocence. Similarly, a fetus or a neonate at the edge of viability should be medically treated, in an ordinary way, unless there are compelling reasons for not treating it in an ordinary way or even at all.

### Principle of Positive Compelling Reasons

If the parents of a neonate at the edge of viability wish its life and health to be medically protected, then both morally and ethically the neonate should be administered best possible medical treatment required for protection of life and health and enhancing viability.

At the edge of viability, chances of healthy survival of a neonate can be low, in which case the question may arise as to whether efforts should be made in order to save its life and protect its health. The principle of positive compelling reasons answers the question affirmatively, on two different grounds, when the parents of the neonate wish the neonate’s life and health to be protected.

The wish of the parents is a compelling reason under the circumstances. First of all, the wish of the parents is highly significant, on the grounds of general principles of medical ethics. Providing a viable neonate with the best possible medical treatment in order to save its life, if in danger, and improve its health, when in trouble, is on a par with providing the fetus in the uterus the best possible medical treatment, under parallel conditions. In both cases, the duty to provide a neonate or a fetus with such medical treatment is a most natural extension of the duty to provide a person with best possible medical treatment. It is a most natural extension of that duty, because a viable neonate or fetus, even before it develops into a person, is encountered within a developmental process that is bound, if left undisturbed, to cross the threshold of personhood.

Moreover, from the point of view of medical ethics, the wish of the parents is not only significant, but also compelling. During the whole period of a pregnancy, from conception to birth, medical treatment is always provided at the request of the woman, not only to herself, but also to the pre-embryo, embryo, or fetus in her womb. Her wish that the two of them be treated is a compelling reason for providing both of them with the best possible medical treatment, even before the fetus becomes a person, even before the fetus becomes viable. The difference between a fetus at the edge of viability and a neonate at the edge of viability is no ground for a radical change in the physician’s duty according to medical ethics. In a sense, the difference is technical. When in the womb, the woman’s wish that it be treated is, to a significant extent, independent of the wish that she herself be treated. Such an independent wish cannot change its ethical significance when the fetus is removed from the womb into an incubator.

The wish of parents that their viable neonate be given the best possible medical treatment is also morally compelling, even when the neonate is at the edge of viability. Firstly, extending the moral duties of protecting human dignity from persons, including viable neonates of a certain gestational age, to viable neonates several weeks younger, is the best way of protecting human dignity of persons at the border areas of personhood. Secondly, when parents have exercised their rights of procreation in a way that resulted in the existence of a viable neonate, then they ought to be helped in extending the process from the present advanced stage to the later stages, which are naturally most desirable.

### Principle of Withholding Treatment

In the absence of compelling reasons for saving the life of a viable neonate, treatment may be withheld from it, provided that:

(a) some curable aspects of the neonate’s health are treated;
(b) pain of the neonate is eliminated or minimized;
(c) its parents are given appropriate palliative treatment.

We assume that under certain conditions there are no moral or ethical compelling reasons for protecting the life of a viable neonate. An extreme example would be a neonate whose viability is a matter of several weeks and there is no doubt that it is not going to survive for even several months. Another possible example is a neonate who certainly suffers from the Tay–Sachs disease.

Assuming there are no compelling reasons for life-saving treatment, the principle of withholding treatment allows medical treatment to be withheld from the neonate, only under certain conditions.

The first precondition of withholding, according to the principle of withholding treatment, is that withholding treatment that saves life should not take the form of totally neglecting the neonate. Protection of human dignity, whether of persons or viable neonates, cannot involve total neglect, on the part of physicians and others who are in a position to pay respect in a clear and effective way. Medical treatment should go on as if permission to save life and perhaps also to treat some other medical problems has not been given, but permission to treat some present medical problems has been given. Division of medical problems into those that should be treated and those that should not depends on various aspects of particular circumstances, such as the parent’s wishes, but whatever those circumstances are, an apparent total neglect of a neonate should never take place.

The second precondition of withholding is obvious, both morally and ethically. If a neonate suffers, whether it is a person or not, whether it is viable or not, it should be treated in order to eliminate every form of pain or, if impossible, to minimize it to the best of present medical ability. A by-product of a treatment that is intended to alleviate pain is sometimes a reduction of life expectancy. Since under consideration is the case where there are no compelling reasons for saving the life of a neonate, then reduction of life expectancy is consequently permitted, but only if it is a reduction of life expectancy and not setting it at zero. Killing is not a legitimate form of alleviating pain, neither from a moral point of view nor from an ethical one.

The third precondition is also self-evident, even though it is not yet universally followed, for various reasons.

### Principle of Negative Compelling Reasons

*When all of the following conditions obtain, with respect to a neonate at the edge of viability, there are compelling reasons for withholding from it medical treatment for saving its life:*

(a) The neonate is bound to suffer, regularly and significantly;
(b) the chances are nil or very close to it that the neonate grows to become a person who eventually will be able to have a meaningful form of life;
(c) the parents of the neonate do not wish that its life be protected, on grounds of both the neonate’s predicted fate and their own resulting mental predicament.

Under consideration is a certain neonate, at the edge of viability. Presently, and all the more so in the future, being at the edge of viability means not being a person, on the one hand, but being at an advanced stage of a process that is usually expected to bring one into becoming a person, on the other hand. Under such circumstances, the neonate does not have a primary moral right to be medically protected, but there are moral and ethical duties that apply to it and require that it be medically protected, unless there are compelling reasons for withholding life-saving medical treatment.

In order to see the justification we have for taking conditions (a)–(c) to form a compelling reason for withholding life-saving medical treatment from a neonate at the edge of viability, let us consider another situation. Apparently that situation is very different, but under analysis it turns out to be rather illuminating. This is the case of an adult patient, for whom the following conditions obtain:

(a) He has an incurable disease;
(b) he is regularly in significant pain;
(c) he is presently unable to maintain what he considers a meaningful form of life and is also never going to be able to maintain such a form of life;
(d) he is perfectly competent, fully aware of his medical and personal conditions.

Assume that the patient expressed a clear and firm wish not to be medically treated any more. Assume that he was asked to explain his wish. His reply was that he knows that he is unable to maintain a meaningful form of life and that he is never going to be able to maintain such a form of life, and therefore he knows that any life-saving medical treatment is going to be worse than worthless. It is not going to provide him with a precondition for maintaining a meaningful form of life but rather with a precondition for maintaining a meaningless living form of regular and significant pain and suffering. When this is the explanation, withholding life-saving medical treatment would not only be considered mandatory on grounds of lack of informed consent, but would also seem to be morally and ethically justified on grounds of the compelling reasons the patient has for his wish.

A period of regular and significant suffering that does not enable the patient to maintain a meaningful form of life could still be worth going through. This could happen when there is a reasonable hope that the period of suffering be followed by a period during which the patient is going to be able to maintain a meaningful form of life. In the absence of such hope, on grounds of the best medical evaluation of the patient’s condition, extending life is nothing more than extending a precondition for suffering, in which the patient is not interested at all. Since under such conditions there does not seem to be any moral or ethical reason to enforce life-saving treatment on the patient, his wish that such medical treatment be withheld from him is a compelling reason for fulfilling this wish.

The situation of a neonate, at the edge of viability, for whom conditions (a) and (b) of the principle of negative compelling reasons obtain, is on a par with that of that patient, *mutatis mutandis*. To be sure, there is a major difference between the adult patient of our example and a neonate at the edge of viability. Whereas the former has a view of what he means by maintaining a meaningful form of life, the latter cannot have such a view. Moreover, the view of the neonate’s parents as to what is a meaningful form of life pertains just to the two of them and as such has no compelling validity with respect to the neonate’s future possible forms of life. In order to overcome the inherently subjective nature of answers to questions with respect to meaningfulness of possible forms of life, condition (b) of the principle of negative compelling reasons is carefully phrased. It is couched in terms of *a meaningful form of life* in general, and not in terms of *a meaningful form of life* according to the views of the neonate’s parents. Consequently, in order to show that condition (b) obtains, it is not enough to show that a certain form of life that is commonly held to be meaningful could not be maintained under the circumstances medically predicted for the neonate. What should be shown is that any form of life that can reasonably be held to be meaningful could not be maintained under those circumstances.

Condition (c) of the principle of negative compelling reasons also requires some clarification. In the absence of a wish on the part of the neonate’s parents that it be medically treated, there are no positive compelling reasons for treatment, and the gate is open for negative reasons and possibly negative compelling reasons for not applying to the neonate any life-saving treatment. The same condition requires that parents have two reasons for not having a wish to protect the neonate. The two conditions are required rather than just one of them, because, for a principle that allows withholding life-saving treatment from a viable neonate to be compatible with moral and ethical principles of protecting human life and dignity, it should be confined to extreme cases.

The condition requires that the attitude of the parents be shaped not only by self-centered considerations, but also by considerations related to the predicted fate of the neonate. Given the proximity of the state of being a neonate at the edge of viability to that of being a person, it would be morally unacceptable to opt for withholding life-saving treatment on parental self-centered grounds only. Parents are morally expected to be the party most devoted to and responsible for the fate of an offspring, at least as long as it is not an independent person.

The condition requires that the attitude of the parents be shaped not only by considerations related to the predicted fate of the neonate, but also by considerations that pertain to the predicament of the parents themselves. If they have the stamina to carry the burden of supporting a suffering baby, who does not have any prospects of becoming a person who can maintain a meaningful form of life, then they are going to be admired and praised. However, their condition cannot then count as an ingredient of a negative compelling reason.

Now, that we have seen the principle of positive compelling reasons as well as the principle of negative compelling reasons, the natural question arises as to whether the two principles exhaust all possible or even prevalent conditions under which neonates at the edge of viability are encountered. The answer is clearly in the negative.

When under consideration is a neonate at the edge of viability, for whom there are neither positive compelling reasons for medical treatment nor negative compelling reasons for withholding life-saving treatment, both principles should serve as sources of guidelines for morally and ethically proper decision-making. Roughly speaking, the distance, so to speak, between the conditions under consideration and those of the pole of positive compelling reasons, on the one hand, and those of negative compelling reasons, on the other hand, should be evaluated. Action should be taken on grounds of the emergent balance of distances.

In case of a remaining doubt or hesitation, one should prefer the attitude of the positive pole to that of the negative one. One defense of this preference rests on comparison of errors and their effects. If one commits an error and treats a neonate that should not have been treated, the results are possibly an unnecessary pain and a reversible general condition. However, if one commits an error and does not treat a neonate that should have been treated, the result is probably death, which is an irreversible general condition. When in such a doubt, then, opting for treatment rather than for withholding is the right preference.

## MATTERS OF COST

So far, we have hardly mentioned expenditure considerations, when treatment of neonates at the edge of viability has been discussed. It is our view that moral and ethical deliberations should always involve two steps: First, discussions of the basic problems and conflicts, principles and attitudes, disregarding restrictions imposed on certain contexts of treatment for financial reason; and, secondly, discussions of the different ways of implementing the consequences reached in the previous step, taking into account the given financial restrictions.

To grasp the reason for our view that deliberations should take such a two-step form, consider the simple case of a physician who has to administer some medication to a sick person, within the framework of some health care service. The seemingly simple and ordinary interaction of prescribing certain pills may, actually, take one of two forms that are morally and ethically different from each other. One way of doing it would involve a list of pills the physician is allowed to prescribe to the patient. The physician would prescribe for the patient the best pills on the list. For financial reasons, the list does not include all available pills. It is quite possible that what is the best pill the physician could have prescribed to the patient under the circumstances is not on the list. Presumably, the physician knows about such pills but neither prescribes them nor informs the patient about their availability elsewhere. In a variant of this procedure, if the physician knows that better pills are available elsewhere, this fact is brought to the attention of the patient.

Another procedure involves a clear distinction between the medical and the financial aspects of the interaction. The physician informs the patient about several possible pills, in the order of their medical value, under the circumstances. The best pill is mentioned, even if it much more expensive than the other ones. The patient gets from the physician a clear description of available treatments, whether within the framework of the service or only elsewhere. Having such a description at one’s disposal, the patient is in a position to make, with the help of the service, a decision as to which pill he or she is going to use. One possibility would be using the best available pill, the purchase of which involving an additional payment. Another possibility would be using another effective pill that is not the best one, but the purchase of which involves no additional payment.

The merits of the latter procedure are clear. Considerations of different kinds are kept separate from each other. The patient’s trust in the physician’s prescriptions can be maintained, as long as it is mutually agreed that financial considerations do not have a hidden effect on the professional quality of the physician’s behavior.

When the treatment of neonates at the edge of viability is to be planned, the same general considerations should be applied. The map of possible treatments should be described, using the standards involved in informed consent. Financial considerations should, then, be superimposed on the medical ones. When the distinction between medical considerations and expenditure considerations is not blurred, the decision made, with respect to issues of life and death, is expected to be of higher moral and ethical qualities.

A crucial consequence of this argument pertains to the very specification of the edge of viability. It follows from our discussion that it is unjustified, both morally and ethically, to withhold treatment of neonates at the edge of viability as a matter of policy on grounds of financial considerations.

Information to the effect that a neonate is at the edge of viability should always be given to its parents. Their wish to protect the neonate’s life and health is a compelling reason for treating the neonate, according to the principle of positive compelling reasons. Deliberations of the financial aspects of the treatment should be carried out after the medical information has been given to the parents who are then given some time for pondering their wishes. Meanwhile medical treatment of the neonate continues. After a while, which may be required to be rather short, the parents make up their mind. It is only at this stage that financial considerations should enter the picture resulting in a distribution of the ensuing expenses. Ideally, on grounds of governmental or other public support and health insurance policies, all neonates at the edge of viability are treated, unless the principle of negative compelling reasons applies. That principle does not make any reference to financial considerations.

## IN SUMMARY

The purpose of the present paper has been to propose and defend moral and ethical principles of medical treatment at the edge of viability, on the grounds of a general conceptual framework. The paper can serve as a starting-point for moral and ethical guidelines both in general and in particular cases under consideration.

